# Exploring the Relations Among Teachers’ Epistemic Theories, Work Engagement, Burnout and the Contemporary Challenges of the Teacher Profession

**DOI:** 10.3389/fpsyg.2022.861437

**Published:** 2022-04-25

**Authors:** Heidi Lammassaari, Lauri Hietajärvi, Katariina Salmela-Aro, Kai Hakkarainen, Kirsti Lonka

**Affiliations:** ^1^Faculty of Educational Sciences, University of Helsinki, Helsinki, Finland; ^2^Optentia Research Focus Area, North-West University, Vanderbijlpark, South Africa

**Keywords:** epistemic theory, teacher, work engagement, burnout, curriculum, digitalization

## Abstract

Current educational reforms concerning curricula and digitalization challenge educators to meet new demands for learning and schooling. What is common for current educational reforms is that they tend to emphasize competencies that are not related to the traditional subject-matters and reflect a stance which presents learning as a naturally reflective and collaborative act. It is often assumed that teachers are automatically ready to implement ideas of this kind in practice. In this study, we propose that teachers’ theories about knowledge, knowing and learning, particularly their epistemic theories, may be related to how teachers approach these reforms which challenge their previous ways of working and how they perceive their wellbeing at work. To examine these matters, we explored the dynamic interrelations between teachers’ epistemic theories, conformity with the novel curricular and digital reforms (ideas behind the new curriculum and digitalization program), perceptions of the school leadership, work engagement and burnout. Participants (Study 1 *n* = 228; Study 2 *n* = 200) were Finnish class teachers and subject-matter teachers. Both data sets were collected before the COVID-19 pandemic. For data analysis, we plotted correlation network figures. Results showed that if teachers’ epistemic theory was in harmony with the curricular or digital reforms, there is a positive association with work engagement and negative association with burnout. In sum, results of this provided a hint of the phenomenon suggesting that teachers’ epistemic theories may be a factor which buffers teachers to meet the current epistemic and developmental challenges of teachers’ profession, and furthermore, serve as grounds for a positive association for teachers to feel adequate and satisfied in their work.

## Introduction

While current policy documents concerning education have started to reflect exceedingly complex theories on the nature of knowledge and learning, new national curricula have been introduced during the last 5 years in several parts of the world followed by the Organisation for Economic Co-operation and Development (OECD) guidelines for 21st century competencies (OECD, 2018). Simultaneously, the overall globalization megatrend has guided schools to take a digital leap and therefore to reconsider the overall conceptions and contexts of learning (e.g., Chiu et al., 2021). In implementing these reforms, teachers play a central role and that is not an effortless task.

Various teacher-related factors may foster or hinder the implementation of curricular requirements, but teachers’ beliefs are the filters, frames and underlying states of expectancy which color teachers’ work, and from a wider perspective, schools’ organizational change (Rokeach, 1968; Fives and Buehl, 2012). Especially teachers’ *epistemic beliefs* (beliefs about knowledge and processes of knowing) are of interest when looking at the ideas teachers put into action (Fives and Buehl, 2012, 2016). That presumption is based on evidence showing that epistemic beliefs may shape and predict which teaching practices and pedagogy are applied (see e.g., Nespor, 1987; Pajares, 1992; Richardson, 1996; Fives and Buehl, 2012, 2016; Buehl and Beck, 2015). It is also indicated that teachers’ epistemic beliefs (or personal epistemologies or conceptions of knowledge and learning, as they were referred to at the time) were related to their ideas about how to foster learning of their students (Lonka et al., 1996; Hofer and Pintrich, 1997). It has been also pointed out that teachers’ beliefs about learning and teaching could even become obstacles to instruction, when teachers are overly relying on their intuitive laypeople theories, for instance, teaching and learning as non-problematic processes that can be learned by experience only (Joram and Gabriele, 1998). However, epistemic beliefs are not only separate beliefs about the nature of knowledge and knowing, but together they construct coherent *epistemic theories* (Hofer, 2016; Lonka et al., 2021).

In the present article, we explore Finnish teachers’ epistemic theories and their perceptions of the Finnish National Core Curriculum (Finnish National Agency for Education, 2014) which underlines the development of broad-based competencies and interdisciplinary projects that cross the boundaries of subject-matter learning (Lonka, 2018). In general, implementing the new national curriculum reflected the growing trend of emphasizing 21st century competencies and active citizenship, as well as calling for metacognitive skills and collaborative knowledge construction was something new, even reformative (European Parliament, 2015). After this curricular renewal there was a pressure to implement a more detailed digital strategy to implement digital tools and digital learning practices better into schooling in 2019. Related to this, we then studied how teachers’ epistemic theories are associated with their approaches to new digital demands.

Adopting pedagogical ideas that are not based on traditional subject-matter teaching and schooling practices and bringing them into the classroom may fundamentally challenge teachers’ intuitive epistemic theories, as they need to rethink the basis of their ideas about what learning is and how it should be promoted. Therefore, we are also interested in how challenging teachers’ epistemic theories are related to teachers’ wellbeing at work, especially work engagement and burnout. There is evidence indicating that shared and congruent beliefs about the importance of desirable end states or behaviors relate to job satisfaction and organizational engagement (Edwards and Cable, 2009). We focused on work engagement that typically manifests itself as high dedication, vigor, and absorption at work (Schaufeli et al., 2006). It is important that the work is experienced as meaningful and aligned with the teachers’ state of expectations and values. If there is a match between teachers’ epistemic theories and the ideas behind the reforms, it may promote work engagement.

In turn, if occupational stress is prolonged and gradually becomes chronic, it may lead to burnout (Maslach et al., 2001; Schaufeli et al., 2002; Salmela-Aro et al., 2011). There also is evidence that a lack of social or administrative support or a lack of coherence between the teacher and the working environment may be the kinds of stressors that pose a risk of burnout (Verquer et al., 2003; Cable and Edwards, 2004; Pillay et al., 2005; Kokkinos, 2007; Sharplin et al., 2011). Recently, there have been many other challenges in schools even before the advent of the COVID-19 pandemic, such as financial cuts, a refugee crisis and an ambitious inclusive approach in Finnish schools: there can be students from a range of socio-economic and ethnic backgrounds as well as special needs students in the same class (Lonka, 2018).

To examine these matters, we studied the dynamic interrelationship between teachers’ epistemic theories, conformity with the new curriculum and digital demands (ideas behind the new curriculum and digitalization program), perceptions about pedagogical leadership, work engagement and burnout. We investigated the correlation networks in two different datasets that helped us to point out the connections between these variables. These matters were first studied at the time when the new curriculum was introduced (2016; Study 1). Then we studied the same matters 4 years later, except the main challenge was no longer implementation of the new curriculum, but the new strategy for successful digitalization process in Finnish schools (2019; Study 2). We assumed that if teachers’ epistemic theories were in line with the ideas behind the curriculum reforms, it would be manifested as conformity in implementing the new practices, and moreover, it would relate to higher work engagement and lower rates of burnout. Moreover, it was presupposed that major changes in teachers’ work call for an empowering or servant kind of leadership (Upadyaya et al., 2016).

### Teachers’ Epistemic Beliefs Constitute Epistemic Theories

Teachers maintain a range of beliefs about knowledge and knowing. These beliefs are considered to be *epistemic beliefs*, and they are independent beliefs about knowledge, especially about the nature of knowledge and knowing, and how knowledge can be acquired and justified (see e.g., Schommer, 1990; Hofer, 2000, 2004). Epistemic beliefs have links to several facets of knowledge-related functioning. Schommer (1990) and Lonka and Lindblom-Ylänne (1996) showed that students’ epistemic beliefs were closely related to their conceptions of learning (see also Lonka et al., 2021). Further, when investigating teachers’ epistemic beliefs, they have shown to be closely connected with teachers’ beliefs about teaching and learning (Maggioni et al., 2009; Olafson and Schraw, 2010). Epistemic beliefs also have a practical dimension: among teachers, epistemic beliefs are related to their instructional practices, such as how they help students in acquiring knowledge, building understanding on learning, and seeking for the truth (Nespor, 1987; Pajares, 1992; Hofer, 2004; Tsai, 2007; Maggioni and Parkinson, 2008; Vedenpää and Lonka, 2014; Buehl and Fives, 2016).

Epistemic beliefs constitute systematic dimensional constructs, namely, epistemic theories (Hofer, 2016; Muis et al., 2016; Lonka et al., 2021) that are also as an essential part of broader cognitive construction referred to as *epistemic cognition* (Chinn et al., 2011; Hofer, 2016; Lonka et al., 2021). This term is holding a position as an umbrella term for various kinds of cognitive processing related to epistemic matters (e.g., Greene et al., 2016; Hofer, 2016). The term *personal epistemology* has also been used (Hofer and Pintrich, 1997), but as terms, epistemic cognition and epistemic beliefs have recently been the most predominant (Sinatra, 2016), especially epistemic cognition holding a position as an umbrella term for various kinds of cognitive processing related to epistemic matters (e.g., Greene et al., 2016; Hofer, 2016). Common in epistemic beliefs and epistemic theories is their multidimensional nature meaning that separate beliefs constitute belief systems which may or may not develop in a synchronized way (Schommer, 1990, 1993; Hofer and Pintrich, 1997; Hofer, 2004). This multidimensional approach for epistemic beliefs can be distinguished from other approaches such as developmental or contextual approach which represent a slightly different perspective on epistemic beliefs (see e.g., Hofer, 2016).

On the basis of work by Lammassaari et al. (2021) and Lonka et al. (2021), the present article explores two quite general dimensions of teachers’ epistemic theories that were confirmed both in Finnish and in Taiwanese context: *the knowledge transmission theory* and *the reflective-collaborative theory*. The former sees knowledge as certain and simple in nature, referring to the process of knowing as mere transmission, and the justification of knowledge being based mostly on certain facts delivered by the teacher. The latter epistemic theory presents knowledge as complex, relativistic and integrated in nature, and the process of knowing is creative, constructive and collaborative, and the source of knowledge is mostly based on scientific references and methods, which can be critically reflected by applying metacognitive processes. Whereas the knowledge transmission theory is often manifested as passive reproduction [e.g., the review by Fives et al. (2015)], the reflective-collaborative theory could be seen more as active knowledge construction (Lonka, 1997; Deng et al., 2014). As these two kinds of theories may occur rather clearly, they are not necessarily opposite (Lammassaari et al., 2021). In higher education, such theories have also been referred to as *the student-centered approach*, aimed at facilitating students’ learning processes and knowledge construction, and *the teacher-centered approach* which emphasizes a way of teaching in which students are more like passive recipients of information that transmitted from the teacher to the students (e.g., Trigwell and Prosser, 1996a,b; Kember and Kwan, 2000; Postareff and Lindblom-Ylänne, 2008). These approaches may be field-related: recently Lonka et al. (2021) showed that pre-service teachers were more likely to express the reflective-collaborative epistemic theory than higher education students from other fields. Less is known about how teachers’ epistemic theories are in line with the novel challenges of their profession (e.g., implementing new curricula or demands related to digitalization), and how it is related to teachers’ wellbeing at work.

### Curricular and Digital Reforms in Schools

In addition to the traditional subject-matter content, new, modernized curricula around the globe reflect new learning-related aims as they have begun to introduce content such as transversal competencies and interdisciplinary learning to be implemented in education at all levels (Lonka, 2018; OECD, 2018). Alongside the nation-specified curricular work, the OECD as a global, widely acknowledged policy forum has notably had its impact on the future of education. As an example, the OECD’s Learning Compass (OECD, 2019) is a framework which presents not only shared language for international education-related discussion, but also a broad vision for the future of learning and desired competencies. It expresses “the need for students to learn to navigate by themselves through unfamiliar contexts and find their direction in a meaningful and responsible way, instead of simply receiving fixed instructions or directions from their teachers” (OECD, 2019, p. 24). These agency-related and competence-related ideas come alive in schools when constructivist, collaborative and reflective learning activities are required. However, it seems that implementing such inherently complex ideas into teaching is more difficult than the educational community tend to expect (Windschitl, 2002). Moreover, it has been shown that although teachers may express the desire to maintain nuanced epistemic beliefs and theories in harmony with the epistemic ideas of the new curricula, their actual classroom practices could continue to be quite traditional (Hofer, 2001; Vedenpää and Lonka, 2014; Buehl and Fives, 2016).

In the ideal situation, technology use should co-evolve hand-in-hand with novel learning and teaching practices (Hakkarainen, 2009; Lonka, 2018). Before the COVID-19 pandemic, the overall digital transformation in schools tended to be inert, the reason probably being that using digital technologies successfully in education call for fundamental changes in the knowledge practices in schools and other institutions (Hakkarainen, 2009). At present, collaborative tools and data-enriched technologies are increasingly about to be adopted in education (OECD, 2018), but that still requires both strong developmental upbeat from teachers, and readiness to review critically one’s own beliefs, expectations and conceptions about learning.

Becoming a modern learning environment, various practices to enable both individual and collaborative professional growth must be adopted. According to Vaara and Lonka (2014), this requires dynamic pedagogical leadership which occurs in schools mostly at the interface between the traditional domains of classroom activities and formal administration. Such leadership is often characterized by empowerment and accountability, namely servant leadership (Russell and Stone, 2002), and it creates opportunities for employees, helping them to grow. This kind of leadership promotes work-specific and general wellbeing but also seems to buffer against the negative impact of workload (Luthans, 2002; Luthans and Avolio, 2003; Upadyaya et al., 2016) which might appear especially during reform situations which fundamentally change the basic principles of one’s work.

### Wellbeing at Work

In the present study, wellbeing at work was approached through work engagement and burnout. Work engagement is a positive state of mind that reflects one’s experience about one’s work (Schaufeli et al., 2002; Bakker et al., 2011). According to Schaufeli and Bakker (2004), engagement is a multidimensional factor which reveals to how vigorous, dedicated and absorbed teachers felt while working. Energy means having the will to invest mental resources in work, including persistence, which also helps to work resiliently in challenging situations and environments. Dedication is a dynamic combination of enthusiasm, sense of meaning, and deep devotion about work. For its part, absorption reflects deep concentration and attraction to work. Energy and dedication are often considered to be the key elements of work engagement, whereas it is disputed whether absorption is more like the third key dimension of work engagement or rather a consequence of it (Bakker et al., 2008).

While experiencing work engagement, there appears to be a positive individual-workplace relationship which is beneficial for both the organization and the individual (see e.g., Macey and Schneider, 2008; Bakker, 2011; Eldor, 2016). There is evidence that work engagement is related to not only individual proactivity but also how innovative the working community is (see e.g., Hakanen et al., 2008). Work engagement is typically a stable state which makes the difference to the experience of flow that is linked to a certain situation or context, for instance (Schaufeli et al., 2002; Bakker, 2011). Still, work engagement doesn’t appear in a vacuum: both work-related resources and personal resources are factors that relate to how the individual experiences their work (Bakker and Demerouti, 2007; Luthans et al., 2008; Hakanen et al., 2011). Work-related resources might be physical resources, social environment and support for personal professional growth, and personal resources such as individual abilities, competencies and resilience. According to Bakker and Demerouti (2008), these two types of resource, independently or in combination, predict work engagement.

In teachers’ work, its independent nature, sense of competence and support from colleagues and school leaders are found to be such organizational resources that are positively related to work engagement (Rosenholtz and Simpson, 1990; Coladarci, 1992). While personal resources are presented as being positive self-evaluations linked to sense of ability to control and impact on one’s professional environment (Xanthopoulou et al., 2009), we propose that one personal resource in teachers’ work might be their approach to learning in general. First, referring to processes and outcomes related to teachers’ own learning, teacher learning has been found to be a factor that is valuable, especially during school reforms (Bakkenes et al., 2010). In contrast, if teachers’ intentions and epistemic theories are not in line with the demands of the new curriculum, their readiness to change their thinking and practices may hinder. Second, there is evidence in the student-context that appreciating a certain kind of learning (in this case, *reflective* learning) was positively related to study engagement (Heiskanen and Lonka, 2012). Lonka et al. (2021) also found that students who maintained collaborative-reflective epistemic theory expressed higher levels of study engagement, and furthermore, they were more likely to be student teachers than, e.g., science or engineering students. Based on these findings, it seems that in different academic cultures, a certain type of epistemic theory may create a positive relationship to engagement, especially if it is shared institutionally and in the community at hand (see also Lonka et al., 2019).

Job-related burnout, in turn, is a psychological, gradually developing syndrome in response to prolonged stress on the job (Maslach and Leiter, 1999; Maslach et al., 2001). It manifests itself as symptoms of exhaustion, cynicism, and professional inefficacy (see e.g., Maslach et al., 2001; Maslach and Leiter, 2005; Hakanen et al., 2006) or alternatively sense of inadequacy (Salmela-Aro et al., 2011). Exhaustion refers to lack of emotional energy, feelings of tension and particularly chronic fatigue caused by overstraining work while the second symptom, cynicism, is a detached or a distant attitude about work and a disaffected attitude to the people with whom one works, leading to low organizational commitment and feeling that work has lost its meaning (Maslach et al., 2001; Schaufeli and Buunk, 2003; Hakanen et al., 2006). The third symptom, lack of professional efficacy or sense of inadequacy, refers to a sense of incompetence and feeling of lacking successful achievement and productivity at work (Hakanen et al., 2006; Salmela-Aro et al., 2011).

How burnout evolves has been widely discussed. Previous studies in work contexts have shown that time pressure, work overload, emotional burden and lack of social or administrative support are all positively correlated with burnout symptoms (Schaufeli and Bakker, 2004; Hakanen et al., 2006; Kokkinos, 2007). Moreover, the lack of congruence and accordance between an employee and the working environment have been found to increase stress and job dissatisfaction, and therefore produce an increased risk of burnout (Pillay et al., 2005; Sharplin et al., 2011).

In comparison to other academic client-related professions, the average level of experiencing burnout symptoms among teachers has been found to be comparably high (Maslach et al., 1996; Schaufeli and Enzmann, 1998). Continuous challenging interaction situations, experiencing an intense work pace, heavy workload and an increasing number of administrative assignments are examples of such factors that pose a burnout risk for teachers (Leithwood et al., 1999; Hargreaves, 2003; Lindqvist and Nordänger, 2006; Skaalvik and Skaalvik, 2010). Also, it has been found that prolonged unsolved problems and perceived destructive frictions within the professional community play a role in the stage of burden that is experienced, gradually leading to the development of teacher burnout (Pyhältö et al., 2011).

Teacher burnout is not only an individual matter, but it might also have its dynamic and complex reflections to the working context in hand. Retelsdorf et al. (2010) found that teacher burnout is related to an increased use of performance-oriented teaching practices. In turn, they are related to lower improvement in the students’ conceptual application skills (Fraser, 1998; Gillies and Ashman, 2003). Moreover, Smylie (1999) showed that teachers’ burnout was inversely related to schools that have been classified as innovative and learning-oriented working communities. In contrast, if individuals who should adopt something new and simultaneously have a strong belief in certain and stabile facts, they may cling to their prior knowledge and focus only on things that support what they already know (Schommer-Aikins, 2011). In the teacher context, this could lead to being incapable of the cognitive flexibility that educational reforms inevitably call for. In this study, we assume that teachers’ epistemic theories may color the way they perceive the novel epistemic aims of obligatory curricula and new digital demands, and therefore how much burden teachers express in their work – manifesting as burnout.

### Context of the Study

This study was carried out in Finland, where the National Core Curriculum for Basic Education (Finnish National Agency for Education, 2014) is an obligatory document for “basic education” (classes 1–9) nation-wide. The National Curriculum of Basic Education (Finnish National Agency for Education, 2014) came into force in 2016, and it introduced a requirement to implement a set of broad-based competencies, as well as requiring regular projects that bridge across the borders of subject-matter domains to be carried out. Simultaneously, the Finnish National Agency for Education (2015), which included similar ideas about learning and competencies was about to be implemented. This curricular content and requirements are created by a group of municipal officials, scholars and experts, and they also guide local curricula development. The core goal for this curricular reform was to strengthen students’ own activity and to increase the sense of meaning of content overall (Finnish National Agency for Education, 2014, 2015). Both the learning goals and learning content were revised to bring them into line with the competence and knowledge requirements of a modern society. In the Finnish National Agency for Education (2014) and for Finnish National Agency for Education (2015) this was manifested as referring to the importance of learning to learn, and as a demand to apply collaborative practices, creative and critical thinking, self-regulatory skills, continuous evaluation of one’s own learning and meaningful use of information technology in instruction. In this study, we operationalized the new curricular demands to call for reflection and collaboration, whereas more traditional ways of instruction based on teaching certain and simple facts would be an aim that is contradictory to the ideals of the current curriculum. Our first data set was collected in 2016, just after the new curriculum implementation process was started.

Technology-enhanced pedagogy has been actively put into practice hand in hand with curricular reform of the Finnish basic education. The prevalent basic education curriculum (Finnish National Agency for Education, 2014, p. 29) makes it imperative for information technology to be “an essential part of multifaceted learning environments” and “novel solutions related to information technology will be adopted to enhance and support learning.” Our second data set was collected in 2019, when the new, municipality-specified digitalization program aiming at bringing digitalization into the core of learning and education was supposed to be implemented and applied in schools that are represented in our data (City of Helsinki Education Division, 2015). Besides enhancing students’ future skills and updating the concept of learning environment, one of the key goals of the digitalization program was to develop teachers’ pedagogical and digital competencies. Another was to bring change management to school principals (City of Helsinki Education Division, 2015, 2016). In this study, the new digital demands were operationalized to call for teachers’ overall active role in developing their digital pedagogy and use digital tools in teaching.

### Aims

In this study, we explored the relations among teachers’ epistemic theories, contemporary challenges of teachers’ profession and wellbeing. We aimed to find out how teachers’ epistemic theories are connected to the novel ideas and requirements of modern curricular and digital demands, perceptions of pedagogical leadership work engagement and burnout. On this basis we posed the following research questions:

What are the dynamic interrelationships between teachers’ epistemic theories, work engagement, burnout, and perceptions of pedagogical leadership:

1. In relation to the new curriculum demands, when was the new curriculum introduced in 2016 (Study 1)?
2. With approach to digital pedagogy and demands set for it when new digital strategy was introduced in 2019 (Study 2)?

We expected that teachers who entertained reflective-collaborative theory, coherent with the new curricular and digital demands, would report higher work engagement and lower burnout. In contrast, it was assumed that teachers who entertained an epistemic theory that reflects knowledge transmission would report lower engagement and higher burnout. Based on previous research we assumed that implementing the curricular reforms described above would require dynamic pedagogical leadership (Vaara and Lonka, 2014).

## Materials and Methods

### Participants

#### Study 1

The participants (*n* = 228) were teachers from two major cities and one town in North-East area of Finland. Participants were teaching in comprehensive schools as class teachers or subject-matter teachers in middle school or upper secondary school. The participants represented a range of subject-matter domains and were at various stages of their career.

#### Study 2

The participants (*n* = 200) were class teachers, subject-matter teachers, and special education teachers in a major city in Finland. They were working either in a primary school or middle school. The participants represented various subject-matter domains and stages of their career.

### Procedure

#### Study 1

The data for the Study 1 were collected by a teacher questionnaire in 2016 as a part of the Mind the Gap study (Academy of Finland, 265528). This convenience sample represented selected schools which participated in the research project (2013–2016). The overall project aim was to examine prevailing gaps between the personal and social practices of students and those of their schools and the educational institutions.

#### Study 2

The data for Study 2 were collected by a teacher wellbeing questionnaire in 2019 in selected six schools as a part of the ongoing Growing Mind study (Strategic Research Council of the Academy of Finland, 312527). These six schools were chosen to be a part of this cooperative project aiming at producing developmental activities related to the aims of the new core curriculum and teachers’ professional development.

In both studies, the participating schools were contacted by the principal investigators of the project and the teachers at these schools were asked to complete the questionnaire. Both questionnaires were conducted electronically.

Data collections were carried out in line with the ethical guidelines for research (National Advisory Board on Research Ethics, 2019), basing the participation on volition, ensuring participants’ anonymity of participants, as well as storing and handling the data according to the ethical permission obtained from the University of Helsinki.

### Instruments

#### Instruments for Study 1 and Study 2

*Teachers’ epistemic theories* were measured with 12 questions that first posed the same item at the idea level (A), and then at the practical level (B) (Vedenpää and Lonka, 2014; see an example in Table 1) on scale 1–6 (1 = totally disagree; 6 = totally agree). Their instrument was based on the MED NORD questionnaire (Lonka et al., 2008), which was originally developed for assessing medical students’ conceptions and orientations to knowledge and learning. The original version consisted of 93 items, and later the briefer versions were formulated based on the original version. In this study, four scales were used valuing metacognition, collaborative knowledge construction, certain knowledge, and simple/surface learning. Each scale consisted of three items. As in our more recent study, the first two scales formed *reflective-collaborative theory* and the last two formed *knowledge transmission theory* (Lammassaari et al., 2021). Reflective-collaborative theory emphasized teaching as a process to promote metacognition and collaborative knowledge construction, whereas knowledge transmission theory brought together the dualistic epistemic beliefs emphasizing the certainty of knowledge and seeing teaching as a knowledge transmission process.

**TABLE 1:** Instruments.

Scale name	Scale	No. of items	Abbreviation	Example item	Study 1	Study 2
Reflective-collaborative theory	1–6	6	RCT	*(A) In my opinion, it is essential that students generate new ideas and thoughts together* *(B) I reserve a lot of time for this in my work as a teacher*	x	x
Knowledge transmission theory	1–6	6	KTT	*(A) The main aim of teaching is to offer certain facts about the subject of study* *(B) This is essential in my teaching*	x	x
Work engagement	1–7	9	EDA	*I find the work that I do full of meaning and purpose*	x	x
Burnout	1–6	9	BBI	*My expectations of my job and of my performance have been reduced*	x	x
New curriculum demands	1–6	6	NCD	*In my opinion, it is essential to develop students’ critical thinking*	x	
New digital demands	1–5	8	NDD	*I strive to develop myself professionally in digital pedagogy skills*		x
Empowering leadership	1–7	7	LEAD	*Leadership offers me abundant opportunities to learn new skills*	x	
Servant leadership*	1–5	4	LEAD	*The principal encourages me to come up with new ideas*		x

*This construct consists of four dimensions of servant leadership.

*Work engagement* was measured by the short Utrecht Work Engagement Scale with nine items (UWES-S; Schaufeli et al., 2002, 2006). The nine items included the following three factors: vigor, dedication, and absorption. The responses were rated on a 7-point scale (0 = never; 7 = daily). Previous research had suggested the use of an overall measure of work engagement (Schaufeli et al., 2006), so we measured the participants’ overall engagement at work in both studies.

*Burnout* was measured by nine items from the Bergen Burnout Inventory (BBI-9; Näätänen et al., 2003; Salmela-Aro et al., 2011) including the following three factors: exhaustion at work, cynicism about the meaning of work and sense of inadequacy. The responses were rated on a 6-point scale (1 = strongly disagree; 6 = strongly agree). Previous research has supported the use of an overall burnout indicator (Salmela-Aro et al., 2011) therefore, we measured the participants’ overall burnout in both studies.

#### Contextual Instruments: Study 1

*New curriculum demands* (Study 1) were measured by six items considering the emphasis of teaching critical and creative thinking and providing constant constructive feedback to the pupils (Finnish National Agency for Education, 2014; Lonka, 2018). The responses were rated on a 6-point scale (1 = totally disagree; 6 = totally agree).

*Empowering leadership* (Study 1) was measured by seven items. *The* scale was based on Van Dierendonck and Nuijten (2011) scale, and the responses were rated on a on a 7-point scale (1 = strongly disagree; 7 = strongly agree). The items considered the encouragement, resources, and autonomous approach for one’s work that the school administration provided and favored.

#### Contextual Instruments: Study 2

*New digital demands* were measured by eight items rated on a 5-point scale (1 = never; 5 = very often). The scale was developed as a part of the Growing Mind study (2019–2023) and its questionnaire targeted at teachers. Items consisted of claims related to the participants’ initiative role in digital development projects in their schools and willingness to learn and adopt new digital tools, methods, and practices to develop their pedagogy. The items were formed to be in line with aims of the new digital program implemented in 2019.

*Servant leadership* was measured by four items (Upadyaya and Salmela-Aro, 2020), and the responses were rated on a 5-point scale (1 = strongly disagree; 5 = strongly agree). The scale was based on Van Dierendonck and Nuijten (2011) scale. The items considered the encouragement, resources, and autonomous approach for one’s work that the school administration provided and favored.

### Data Analysis

Identical procedure of data analysis was applied for both Study 1 and Study 2. First, we specified and tested the structure of the model using item parcels of the A and B parts of the items for the epistemic theories and the raw items for the other constructs. We followed the next fit indices: chi-square, root mean square error of approximation (RMSEA) with an approximate cutoff value for a good fit of less than 0.05, SRMR with a cut-off value <0.08, comparative fit index (CFI) with a cutoff value of greater than 0.96 as well as the Tucker-Lewis index (TLI) with a cutoff value of greater than 0.95 (Hu and Bentler, 1999; Yu, 2002).

The model was specified as a confirmatory factor analysis model (CFA) with ordered items. All items were allowed to load on their corresponding factor only. The analyses were conducted using “lavaan” (Rosseel, 2012) in R and RStudio (RStudio Team, 2020). We used robust weighted least squares (WLSMV) as the estimator. Second, we used a method of visualizing the correlations between the constructs in the model (see e.g., Sjöblom et al., 2020).

To describe the method more specifically, the correlations among the epistemic theories, new curriculum demands, and approach to digital pedagogy, pedagogical leadership as well as work engagement and burnout were visualized and examined. We did this by exporting the latent variable correlation matrix of the model and visualizing the cross-sectional correlations by plotting the latent variables as nodes in a correlation network (Epskamp and Fried, 2018). We used R-package “qgraph” (Epskamp et al., 2012), similarly to a latent variable network model (see e.g., Epskamp et al., 2017).

The edges in the latent correlation network represent simple bivariate correlations. The figures display the strength of correlations between the different constructs as well as whether it is negative or positive. The strength of this modeling is that it allows for powerful measurement error-corrected modeling of undirected structural relationships between latent variables (Guyon et al., 2017).

## Results

Table 2 shows means, standard deviations and Cronbach’s alphas for variables used in this study.

**TABLE 2:** Raw M, SD, and α for Study 1 and Study 2.

	Study 1			Study 2		
	Raw M	SD	α	Raw M	SD	α
Reflective-collaborative theory	4.8	0.619	0.887	4.9	0.494	0.828
Knowledge transmission theory	3.3	0.903	0.901	3.5	0.656	0.832
Work engagement	6.1	0.854	0.898	5.4	0.892	0.915
Burnout	2.4	0.969	0.861	2.6	0.887	0.860
**Contextual measures**						
New curriculum demands	4.5	0.691	0.776			
New digital demands				3.3	0.720	0.879
Empowering leadership	5.0	1.43	0.929			
Servant leadership				3.5	0.662	0.608

The model in Study 1 was a good fit for the data [chi-square (725) = 1214.32, scaling = 1.837, *p* < 0.001, RMSEA = 0.055, SRMR = 0.081, CFI = 0.947, TLI = 0.942]. The model in Study 2 also fit the data well enough [chi-square (804) = 1324.53, scaling = 2.366, *p* < 0.001, RMSEA = 0.065, SRMR = 0.095, CFI = 0.937, TLI = 0.932], and no *post hoc* modifications were considered for the sake of parsimony and replicability.

### Study 1

**FIGURE 1:**
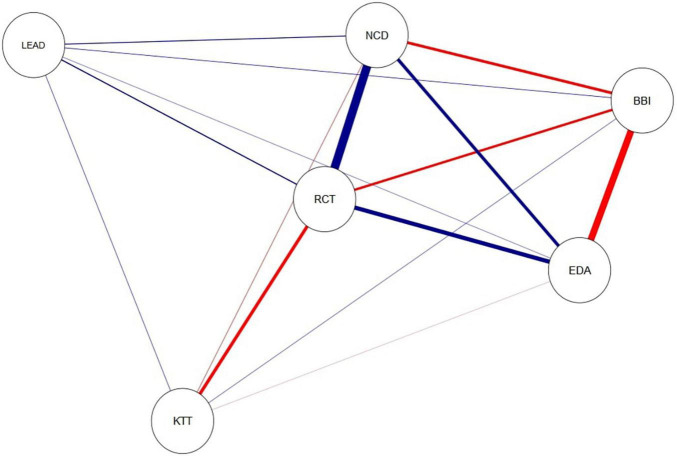
Latent variable correlation network Study 1. Abbreviation RCT refers to reflective-collaborative theory, KTT to knowledge transmission theory. Abbreviation NCD (NCD, new curriculum demands) refers to items measuring the conformity with the new curriculum demands, LEAD to empowering leadership, EDA to work engagement, and BBI to burnout symptoms. Blue edges in the figure refer to positive relations, and red edges to negative relations. The width of the edges corresponds to the absolute value of the correlations: the higher the correlation, the thicker the edge (see Epskamp et al., 2012).

We studied the relationships between the epistemic theories and new curriculum demands, empowering leadership, work engagement and burnout. The model seen in Figure 1 indicated that there were direct positive relationships between reflective-collaborative theory, conformity with the new curriculum demands (0.75), work engagement (0.40), and empowering leadership (0.15). This was in line with our expectations. Reflective-collaborative theory also mediated a negative relationship between knowledge transmission theory and burnout. In turn, teachers’ knowledge transmission theory was positively related to empowering leadership (0.09) and burnout (0.07), but negatively to conformity with the new curriculum demands (−0.08) and work engagement (−0.03). The relationships between knowledge transmission theory and the other variables were mostly expected; however, except for a negative relationship between the two epistemic theories, the relationships were quite modest, as the width and frail edges between these variables indicate in Figure 1.

Then we looked at the results from the perspective of wellbeing variables. Figure 1 shows that teachers’ work engagement had a direct positive association to reflective-collaborative theory (0.40), conformity with the new curriculum demands (0.75), and empowering leadership (0.15). In contrast, work engagement was negatively related to knowledge transmission theory (−0.03) and burnout (−0.60). When observing teachers’ burnout, results show that it was positively related to knowledge transmission theory (0.07) and empowering leadership (0.10), but negatively related to reflective-collaborative theory (−0.25), new curriculum demands (−0.28) and work engagement (−0.60). These findings were mostly in line with our presumptions regarding the coherence between a certain kind of epistemic theory and new curriculum demands, and its reflection on better occupational health.

### Study 2

**FIGURE 2:**
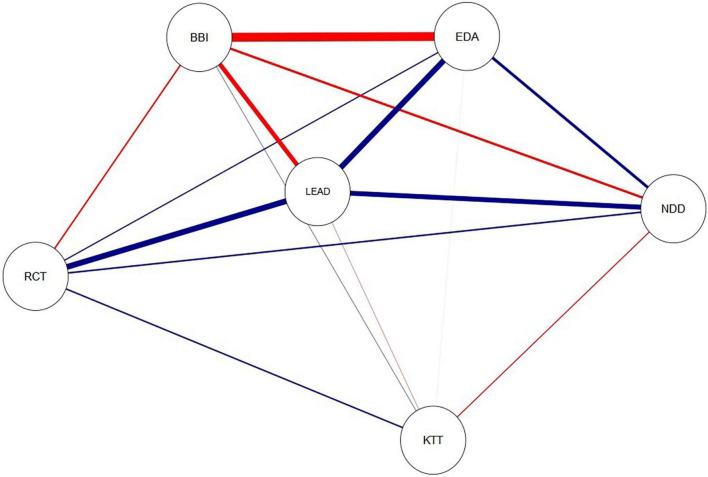
Latent variable correlation network Study 2. Abbreviation RCT refers to reflective-collaborative theory, KTT to knowledge transmission theory, NDD (New digital demands) to developmental orientation for digital pedagogy, LEAD to servant leadership, EDA to work engagement, and BBI to burnout symptoms. Blue edges in the figure refer to positive relations, and red edges to negative relations. The width of the edges corresponds to the absolute value of the correlations: the higher the correlation, the thicker the edge (see Epskamp et al., 2012).

We examined the relationships between epistemic theories and new digital demands, servant leadership, work engagement and burnout. As Figure 2 shows, there were expected positive relationships between reflective-collaborative theory and new digital demands (0.17), servant leadership (0.44), and work engagement (0.14). Contrariwise to Study 1, reflective-collaborative theory was also positively related to knowledge transmission theory (0.16). In turn, knowledge transmission theory was positively related to burnout (0.07), but negatively related to new digital demands (−0.13) and servant leadership (−0.06). These relationships were in line with our expectations although especially relationships between knowledge transmission theory and the rest of the variables were quite modest as the width and thickness of the edges in Figure 2 indicate.

When taking a specific look at the wellbeing variables, Figure 2 shows that the associations between work engagement, burnout and the rest of the variables were mostly in line with our expectations: Work engagement had a positive association with reflective-collaborative theory (0.14), new digital demands (0.25), and servant leadership (0.43). Work engagement had an only direct negative association to burnout (−0.65). In contrast, burnout was negatively related to reflective collaborative theory (−0.16), new digital demands (−0.22), and servant leadership (−0.35), but positively to knowledge transmission theory (0.07). In contrast to Study 1, in Study 2, servant leadership was also mediating the associations between reflective-collaborative theory, and work engagement as well as new digital demands. In these two mediated cases, the relationships were more apparent as thicker edges in Figure 2 indicate.

## Discussion

### Discussion of the Results

In the present study, we aimed to find out what kinds of interrelations can be found out between teachers’ epistemic theories, contemporary professional challenges related to novel curricula and digitalization, perceptions of work-related wellbeing and pedagogical leadership. Overall, the findings of this study extend the understanding of the factors which may be associated with the current institutional renewals concerning teachers’ work. In Study 1 (data collected in 2016), we expected that reflective-collaborative epistemic theory would be positively associated with work engagement and to the new curriculum demands. This was due to the novel ideas and practices presented in the new national curriculum which emphasized reflection and collaboration as an essential part of learning and classroom activities. In Study 2 (data collected 2019), we expected that teachers who expressed this same reflective-collaborative epistemic theory would be more willing to develop their digital pedagogy by using modern digital tools and would therefore also express higher work engagement and lower burnout. During Study 2’s data collection the new digitalization program reflecting the background ideas of the national curriculum reforms was about to be fully implemented.

The results in both studies were aligned with these expectations, however, in Study 2 servant leadership seemed to play its role as a strengthener or mediator of associations between reflective-collaborative epistemic theory, approach to new digital demands, and wellbeing. Both studies pointed out that if teachers’ epistemic theory was in harmony with the reforms, there would appear a positive association with work engagement and negative to burnout. This finding is in line with several previous studies (see e.g., Hakanen et al., 2006; Upadyaya et al., 2016) which have suggested that there occur various person-related factors which might be positively associated with teachers’ wellbeing at work.

In sum, results of these studies provided a hint of the phenomenon suggesting that teachers’ epistemic beliefs could be a resource which buffer teachers to meet the current epistemic and developmental demands of teachers’ profession, and furthermore, serve as grounds for a positive association for teachers to feel adequate and satisfied in their work. As epistemic beliefs or theories are suggested to be socially shared, so that the origin of knowledge does not lie only within an individual mind (see e.g., Packer and Goicoechea, 2000), this resource might not occur only in the individual level but even more in a group or community level. In the case of teachers, this community could be for instance the school where they work.

In contrast, teachers’ knowledge transmission theory was assumed to be less likely to agree with ideas of these same curricular and digital reforms. Knowledge transmission theory was also expected to be associated with lower work engagement and higher rates of burnout. Especially in the case of Study 2, when the focus was on digitalization, the expected associations were not that obvious.

However, what was notable were the associations between reflective-collaborative theory and knowledge transmission theory: in Study 1 the association between these two epistemic theories was negative whereas in Study 2 the association was positive. Due to a cross-sectional nature of this study, there is limited capacity to find an explanation for this difference, nevertheless, in 2016, there might have been a more prominent need to make a shift toward the new national curriculum and its comparably sophisticated *epistemic climate* (meaning how the nature of knowledge and knowing is portrayed and perceived in a certain context, such as in classroom practices or curriculum discourses; see e.g., Feucht, 2010; Muis and Duffy, 2013). That shift might have reflected in our Study 1 results as a more observable juxtaposition between the two epistemic theories and their relationships to other variables under this exploration.

In Study 2 (2019), results hinted that the focus has changed from purely epistemic issues toward the actual challenges of digitalization. This may be due to the fact that a profound curriculum reform is more closely linked to epistemic theories than a digital reform, which may be based on various theories. This could also indicate the growing emphasis on *epistemic fluency* meaning that standing behind a certain epistemic stance is more important than one being able to recognize and use several culturally shared ideas about what knowing is and how knowledge should be constructed (Morrison and Collins, 1995): there is a place for details and facts, but also for a comprehensive approach integrating reflective and collaborative learning practices. In this respect, it is appropriate to remark that these two epistemic theories are not automatically opposite by nature, but they may exist side-by-side as Lammassaari et al. (2021) pointed out.

### Methodological Limitations

The present study has some limitations. First, the study was based on teachers’ self-reports, and the sample size in both studies was quite small. Especially Study 1 represented a convenience sample which is a consequence of the wider challenges in data collection related to teacher questionnaires. Over time, it has been a challenge to collect comprehensive teacher data which may be related to the considerable number of questionnaires constantly addressed to teachers in the profession. Moreover, participants in these two studies were different, making the studies present in this study cross-sectional as a design. Studies in the future call for being carried out with larger samples and with longitudinal design. Longitudinal design could reveal a more precise perception of how new institutional demands such as curricular renewals or novel digital programs and their associations to teachers’ epistemic theories are reflected and evolved over time which was not possible in this study’s setting.

Some scale and overall methodological developments are also required. As an example, our results did not indicate yet the directions of the relationships which would have offered another angle to overview this complex phenomenon of teachers’ epistemic theories and their associations with a range of variables. In this study, we were not assuming causality, merely exploring the complex relationships between epistemic theories, work engagement and the epistemic demands of teachers’ contemporary work. The scales used in this study were mostly robust, therefore more detailed perceptions related to teachers’ wellbeing and as mentioned, more refined associations between used variables might have been left out of the network. Despite these promising results about teachers’ epistemic theories and their relationships to current challenges of teachers’ work and occupational health, a more subtle approach should be applied to explore the wider spectrum of the epistemic theories and overlap that potentially exists between them. This may also require a mixed-method approach. Overall, this is a worthwhile working hypothesis and in the future, we will look more closely into these relations and triangulate the results with several representative samples.

### Future Implications

This variable-oriented study identified associations between teachers’ epistemic theories, new curricular and digital demands, school leadership and teachers’ occupational health. Our research revealed that teachers’ epistemic theories, which consist of teachers’ epistemic beliefs and how they report putting these ideas into practice, play a role when looking at dynamic interrelationships between these theories, new demands set for schooling, work engagement and burnout. Previous research has shown that when taking a closer look at teachers’ wellbeing at work, two profiles of Finnish teachers have been identified: engaged (30%) and engaged-burnout (70%) profiles, the latter being still engaged in their work, but already starting to show some symptoms of burnout (Salmela-Aro et al., 2019). Simultaneously, it has been shown that teachers may express quite complex and constructivist epistemic beliefs and theories, but their actual classroom practices may contradict them (Hofer, 2001; Vedenpää and Lonka, 2014; Buehl and Fives, 2016). Some researchers even propose that theories and beliefs about learning should be distinguished more clearly from theories about teaching (Richardson and Placier, 2001; Fives et al., 2015). In the context of this discussion, we suggest that as a combination, the possible discontinuity between teachers’ epistemic theories, novel epistemic aims and demands set for schooling might show up overwhelming for some teachers and manifest, in some timespan, even as symptoms of burnout.

In this sense, novel approaches are needed to offer all teachers an equal opportunity to adopt new curriculum-related and developmental ideas as well as to implement these changes in their practice. As this study indicated, this is not only an individual matter but also a community-level matter related to, e.g., school leadership that, in its best, encourages and supports teachers to evolve and even transform in their thinking and profession to meet the new requirements. However, epistemic change, if looked for, is not a simple mission since unless individuals have a good reason to abandon their beliefs, they will be unlikely do so (see e.g., Bendixen, 2002; Muis and Duffy, 2013). In the teacher context this might be manifested as clinging onto the traditional pedagogical ideas and practices which have governed schools over time and might still offer a sense of something that surely works. For this reason, it is important to note that a prerequisite for epistemic change is that individuals must be able to understand the new beliefs, and consider them to be plausible, so that they can be applied and be fruitful for further inquiry (Pintrich et al., 1993). Interestingly, it has been shown that working in an innovative and future-oriented school is positively related to teachers’ engagement (Rosenholtz, 1989). Although this offers a hint that school communities which constantly evolve and look forward to the future may keep teachers engaged, we still do not clearly recognize the origins of this phenomenon. Therefore, further research is required to dig deeper theoretically into looking at fruitful preconditions for teachers’ epistemic growth to support the readiness for change implementation. The local and global evolution is ongoing, and the post-pandemic era will inevitably bring again new challenges, such as hybrid learning, for teacher profession.

## Data Availability Statement

The data analyzed in this study is subject to the following licenses/restrictions: GDPR regulations were taken into account. All data that might enable the identification of an individual participant was deleted and replaced by a participant number. Requests concerning these datasets should be directed to the corresponding author. Requests to access these datasets should be directed to HL, heidi.lammassaari@helsinki.fi.

## Ethics Statement

The studies involving human participants were reviewed and approved by the Ethical Review Board in the humanities and social and behavioral sciences of the University of Helsinki. The patients/participants provided their written informed consent to participate in this study.

## Author Contributions

HL: leading writer. LH: researcher of the projects, leading data analysis, supervisor of HL, and a contributor to the writing process. KSA: Co-PI of the Study 1 project and a contributor to the writing process. KH: PI of the Study 2 project and a contributor to the writing process. KL: PI of the Study 1 project, supervisor for HL, and a contributor to the writing process. All authors contributed to the article and approved the submitted version.

## Conflict of Interest

The authors declare that the research was conducted in the absence of any commercial or financial relationships that could be construed as a potential conflict of interest.

## Publisher’s Note

All claims expressed in this article are solely those of the authors and do not necessarily represent those of their affiliated organizations, or those of the publisher, the editors and the reviewers. Any product that may be evaluated in this article, or claim that may be made by its manufacturer, is not guaranteed or endorsed by the publisher.
