# Supplementary material for: Exploring the Relations Among Teachers’ Epistemic Theories, Work Engagement, Burnout and the Contemporary Challenges of the Teacher Profession
Source: Front Psychol. 2022 Apr 25;13:861437. doi: 10.3389/fpsyg.2022.861437 (PMC9081881; doi:10.3389/fpsyg.2022.861437)
Supplement: Supplementary file 1 [file Table_1.DOCX]

*Latent factor correlations for Study 1*

|  | Reflective-collaborative theory | Knowledge transmission theory | New curriculum demands | Empowering leadership | Work engagement | Burnout |
| --- | --- | --- | --- | --- | --- | --- |
| Reflective-collaborative theory |  |  |  |  |  |  |
| Knowledge transmission theory | -.32 |  |  |  |  |  |
| New curriculum demands | .75 | -.08 |  |  |  |  |
| Empowering leadership | .15 | .09 | .13 |  |  |  |
| Work engagement | .40 | -.03 | .32 | .06 |  |  |
| Burnout | -.25 | .07 | -.28 | .10 | -.60 |  |
