# Supplementary material for: Exploring the Relations Among Teachers’ Epistemic Theories, Work Engagement, Burnout and the Contemporary Challenges of the Teacher Profession
Source: Front Psychol. 2022 Apr 25;13:861437. doi: 10.3389/fpsyg.2022.861437 (PMC9081881; doi:10.3389/fpsyg.2022.861437)
Supplement: Supplementary file 2 [file Table_2.DOCX]

*Latent factor correlations for Study 2*

|  | Reflective-collaborative theory | Knowledge transmission theory | New digital demands | Servant leadership | Work engagement | Burnout |
| --- | --- | --- | --- | --- | --- | --- |
| Reflective-collaborative theory |  |  |  |  |  |  |
| Knowledge transmission theory | .16 |  |  |  |  |  |
| New digital demands | .17 | -.13 |  |  |  |  |
| Servant leadership | .44 | -.06 | .39 |  |  |  |
| Work engagement | .14 | .01 | .25 | .43 |  |  |
| Burnout | -.16 | .07 | -.22 | -.35 | -.65 |  |
